# Videogames as a Therapeutic Tool in the Context of Narrative Therapy

**DOI:** 10.3389/fpsyg.2016.01657

**Published:** 2016-10-25

**Authors:** Gilbert E. Franco

**Affiliations:** Southern California Seminary, Graduate Behavioral SciencesBonita, CA, USA

**Keywords:** narrative therapy, videogames, therapist, treatment, family therapy

## Introduction

Videogames can have a significant impact on a person's life. Researchers have looked at how videogames impact a person's life ranging from their creative, mental, and physical aspects to even their social and political worlds (e.g., Montani et al., 2014; Sil et al., 2014; Alhabash and Wise, 2015; Bloom, 2015; Bock et al., 2015; Chang et al., 2015; Yeh, 2015; González et al., 2016; Nebel et al., 2016). Videogames in relation to psychotherapy have also been studied and discussed (e.g., Graham, 2014; Fernandez-Aranda et al., 2015; Franco, 2015; Llorens et al., 2015). Franco (2015) discussed the use of family therapy in treating videogame addiction. Graham (2014), in particular, looked at videogame addiction and how to apply narrative therapy to translate videogame skills from online videogames to clients' offline lives. Rather than using family or narrative therapies to treat videogame addiction, it may serve narrative therapists to adopt videogames as a tool in narrative therapy.

Videogames have been used in group therapy to treat individuals with traumatic brain injury (Llorens et al., 2015) and in conjunction with cognitive—behavioral therapy to treat individuals with bulimia nervosa (Fernandez-Aranda et al., 2015). Board games, videos, and art are often used by therapists to enable their clients to address therapeutic concerns. In that same vein, videogames can be integrated into the narrative therapy model as tools that can enable clients to externalize their problems and re-author their experiences.

## Narrative therapy

Narrative therapy was developed by White and Epston (1990) as a method of providing clients a way of viewing their experiences in larger than individual contexts such as cultural or social contexts. In narrative therapy, the role of the therapist is to be a genuinely curious listener. Narrative therapists attempt to create a therapeutic environment that makes room for possibilities and assume that the stories that are authored organizes a client's experience and shapes their behavior.

Narrative therapists want to externalize the problem and this is often done using externalizing questions (White and Epston, 1990). A common goal in narrative therapy is to change the problem-saturated story in order for the client to achieve their preferred narrative (White and Epston, 1990; Freedman and Combs, 1996). In order for a client to change their problem-saturated stories to achieve their preferred narratives, they have to identify them first. That is where videogames can be used as a tool to facilitate this goal.

## Using videogames as a narrative therapy tool

Role-playing games (RPG's) involve players taking the role and story of imaginary characters (Lis et al., 2015). RPG's can include massively multiplayer online role-playing games (MMORPG's; Servais, 2015), traditional pen and paper RPG's (Lis et al., 2015), and single-player videogame RPG's, such as the Elder Scroll series (Kadakia, 2005). While MMORPG's may have detrimental effects on a person's real life social support (Kaczmarek and Drążkowski, 2014), single-player RPG's can increase a person's engagement (Kadakia, 2005).

Single-player RPG's, such as the Elder Scrolls: Morrowind, connect players to a fantasy world. Morrowind, for example, is a commercial open-ended RPG (Kadakia, 2005). Players can create a character of their choice and interact with the open-ended world in different ways. They can create a heroic character that interacts with the world in a socially constructive manner. They can also choose to create a villainous character or a character that is morally ambiguous. In short, clients can participate in stories that they author within the constraints of the game environment.

A narrative therapist can use an RPG to enable a client to explore alternate stories for themselves. They can explore the consequences of their actions via RPG's and can then process these with the therapist in session. For example, a narrative therapist can give a homework assignment to a client whom owns a videogame like Morrowind. The therapist can ask the client to create a character that has a similar story to the client's current dominant story. During the following session, the narrative therapist can process the homework assignment with the client. After processing the results of the homework assignment, the therapist can then assign the client a homework assignment that involves creating a character that reflects their preferred narrative.

## A vignette

Carrie, a 17 year old Hispanic female, presented symptoms of depression. She has never used drugs, but her mother disclosed in a previous session that she found out that her boyfriend smokes marijuana. The mom fears that Carrie will be coerced into using drugs.

During her most recent individual session, Carrie disclosed that she felt disappointed with her life. When the therapist asked what extracurricular activities Carrie enjoyed, she disclosed that she enjoyed dancing ballet, baking, and playing videogames. The therapist then asked whether Carrie played any RPG's and she said that she played Final Fantasy, Super Smash Brothers, and Morrowind. The therapist asked Carrie if she could create a character in Morrowind that was similar to her. The therapist then asked to play that character in the videogame as if it was her and to base all of that character's actions on what she would actually choose to do if it was her in the game world. Carrie said that she would do the homework.

During the next session, the therapist asked Carrie about the homework assignment. Carrie stated that she chose to become a paladin. She reported that she found herself persecuting “skooma addicts.” When asked what “skooma addicts” were, Carrie stated that they were the videogame equivalent of drug users.

## Conclusion

As with any new intervention that is proposed, it is recommended that future studies assess for the efficacy videogames in conjunction with narrative therapy. Videogames have been found to be a viable intervention when used in conjunction with CBT and group therapy. Expanding research and assessing for its effectiveness in narrative therapy approaches can result in therapists having another therapeutic tool that they can use in treatment. Videogames are popular today and continue to increase in popularity (Franco, 2015). It is a multibillion dollar industry. Using videogames as a therapeutic tool can enable narrative therapists to better relate to and reach a broader range of clients.

A limitation to using videogames in the context of narrative therapy is that enabling clients to create preferred narratives may be limited to the rules and context of the videogame. For example, if the videogame, Morrowind, were to be used in treatment the client would be limited to the fantasy world depicted in the game along with its in game rules such as only being able to participate in the jobs and quests offered by the in game characters. On the other hand, using the rules established by the videogame can give the therapist an idea as to how a dominant story imposed by the videogame is similar to what the client is experiencing in real life. In other words if the rules of the game follow similar patterns as those in the client's real life, the therapist can observe how the client creates their story based on those rules.

A possible solution to this limitation is that therapeutic videogames utilizing an open ended world can enable clients to explore a realm of possibilities presented in a purely therapeutic context. A significant amount of the population plays videogames (Franco, 2016) and not using videogames in the context of narrative therapy, or even therapy in general, may not make therapeutic sense. Adopting videogames as tools, much like board games and movies can make a significant impact on our client's lives.

## Author contributions

The author confirms being the sole contributor of this work and approved it for publication.

### Conflict of interest statement

The author declares that the research was conducted in the absence of any commercial or financial relationships that could be construed as a potential conflict of interest.
